# Fluoxetine impairs GABAergic signaling in hippocampal slices from neonatal rats

**DOI:** 10.3389/fncel.2013.00063

**Published:** 2013-05-01

**Authors:** Maddalena D. Caiati, Enrico Cherubini

**Affiliations:** Department of Neuroscience, Scuola Internazionale Superiore di Studi AvanzatiTrieste, Italy

**Keywords:** fluoxetine, immature hippocampus, GDPs, spontaneous action potential-dependent release of GABA, spontaneous firing of principal cells and interneurons

## Abstract

Fluoxetine (Prozac), an antidepressant known to selectively inhibit serotonin reuptake, is widely used to treat mood disorders in women suffering from depression during pregnancy and postpartum period. Several lines of evidence suggest that this drug, which crosses the human placenta and is secreted into milk during lactation, exerts its action not only by interfering with serotoninergic but also with GABAergic transmission. GABA is known to play a crucial role in the construction of neuronal circuits early in postnatal development. The immature hippocampus is characterized by an early type of network activity, the so-called Giant Depolarizing Potentials (GDPs), generated by the synergistic action of glutamate and GABA, both depolarizing and excitatory. Here we tested the hypothesis that fluoxetine may interfere with GABAergic signaling during the first postnatal week, thus producing harmful effects on brain development. At micromolar concentrations fluoxetine severely depressed GDPs frequency (IC_50_ 22 μM) in a reversible manner and independently of its action on serotonin reuptake. This effect was dependent on a reduced GABAergic (but not glutamatergic) drive to principal cells most probably from parvalbumin-positive fast spiking neurons. Cholecystokinin-positive GABAergic interneurons were not involved since the effects of the drug persisted when cannabinoid receptors were occluded with WIN55,212-2, a CB1/CB2 receptor agonist. Fluoxetine effects on GABAergic transmission were associated with a reduced firing rate of both principal cells and interneurons further suggesting that changes in network excitability account for GDPs disruption. This may have critical consequences on the functional organization and stabilization of neuronal circuits early in postnatal development.

## Introduction

Fluoxetine (Prozac) is a selective serotonin reuptake inhibitor widely used for the treatment of depressive disorders (Wong et al., 1974, 1995). According to the “monoamine hypothesis” of depression, this drug would act by enhancing the extracellular serotonin levels and function in particular brain areas targeted by the serotoninergic system such as the frontal cortex and the hippocampus, thought to be involved in the control of emotion, memory, and cognition (Krishnan and Nestler, 2008). However, whether fluoxetine-induced amelioration of mood disorders is exclusively linked to potentiation of serotoninergic activity or requires other neurotransmitters involved in plastic changes of neuronal connectivity is still a matter of debate (Castrén, 2005). Evidence has been provided that in the adult visual system, chronic administration of fluoxetine is able to reinstate ocular dominance plasticity and promote recovery of visual function in amblyopic animals, an effect associated with a BDNF-dependent reduction of cortical inhibition (Maya Vetencourt et al., 2008, 2011, 2012). Recent findings have indeed indicate that fluoxetine affects GABAergic transmission supporting a casual relationship between a dysfunction of GABAergic signaling and depressive disorders (Luscher et al., 2011). Thus, the acute and chronic administration of two major antidepressant, imipramine and fluoxetine, has been shown to impair GABA_A_-mediated perisomatic inhibition mediated by parvalbumin (PV)-positive fast spiking interneurons, and to disrupt γ-oscillations, further suggesting a new mechanism of action of these drugs in controlling mood disorders (Méndez et al., 2012).

At late embryonic, early postnatal stages of development the inhibitory transmitter GABA depolarizes and excites targeted cells in the hippocampus because of an initially high intracellular chloride concentration (Cherubini et al., 1991; Ben-Ari et al., 2012). The depolarizing and excitatory action of GABA, in synergy with that of glutamate is instrumental for initiating synchronized network activity under the form of giant depolarizing potentials or GDPs (Ben-Ari et al., 1989). GDPs, which constitute a primordial form of synchrony between neurons, have been proposed to be the *in vitro* counterpart of “sharp waves” recorded in rat pups *in vivo* during immobility periods, sleep, and feeding (Leinekugel et al., 2002). Calcium transients associated with GDPs are crucial for enhancing, in a Hebbian type of way, synaptic efficacy at emerging glutamatergic (Mohajerani et al., 2007) and GABAergic synapses (Kasyanov et al., 2004).

Recent data indicate that in the immature CA3 hippocampal region, a few early generated GABAergic interneurons are able to affect network dynamics acting as functional hubs to pace the activity of hundreds of cells *via* their extensive axonal arborizations (Bonifazi et al., 2009; Picardo et al., 2011; Allene et al., 2012). In addition, among the large variety of GABAergic interneurons present in the hippocampus (Klausberger and Somogyi, 2008), those generated later from the medial ganglionic eminence, belonging mainly to parvalbumin-containing perisomatic targeting cells (Cossart, 2011), constitute together with cholecystokinin-positive basket cells the main source of spontaneous GABA_A_-mediated synaptic events recorded from CA3 principal cells (Freund and Katona, 2007). The latter express cannabinoid receptor type 1. Spontaneously occurring synaptic activity may summate to reach the threshold for action potential generation thus contributing to GDPs onset (Ben-Ari et al., 2012).

Fluoxetine is the most commonly prescribed antidepressant drug for women suffering from depression during pregnancy and the postpartum period. However, this drug, which being highly lipophilic crosses the human placenta (Heikkine et al., 2002) and is secreted with milk (Davanzo et al., 2011) may exert harmful effects on the development of fetuses or neonates, respectively. These exhibit an increased risk to develop neuro-developmental disorders and to experience behavioral deficits (Nulman et al., 2002; Oberlander et al., 2009). In addition, animal studies have shown that chronic treatment of pregnant rats with fluoxetine alters the offsprings response of the hippocampus to stress (Olivier et al., 2011a,b; Pawluski et al., 2012).

Here, we tested the hypothesis that fluoxetine affects GABAergic signaling during the first week of postnatal life when most of plastic changes occur. We found that the antidepressant reversibly blocks GABA_A_-mediated GDPs in a concentration-dependent way and independently of its effects on the amine reuptake systems.

## Materials and methods

### Ethical approval

All experiments were performed in accordance with the European Community Council Directive of November 24, 1986 (86/609EEC) and were approved by the local authority veterinary service and by SISSA ethical committee. All efforts were made to minimize animal suffering and to reduce the number of animal used.

### Hippocampal slices preparation

Wistar rats were decapitated after being anesthetized with CO_2_. Hippocampal slices were obtained from at postnatal (P) days P2-P6 (day 0 was considered the day of birth) animals using a standard protocol (Caiati et al., 2010). Briefly, the brain was quickly removed from the skull and placed in ice-cold ACSF containing (in mM): NaCl 130, KCl 3.5, NaH_2_PO_4_ 1.2, MgCl_2_ 1.3, CaCl_2_ 2, Glucose 24, NaHCO_3_ 27, saturated with 95% O_2_ and 5% CO_2_ (pH 7.3–7.4).

Transverse hippocampal slices (400 μm thick) were cut with a vibratome and stored at room temperature (20–24°C) in a holding bath containing the same solution as above. After a recovery period of at least 1-h, an individual slice was transferred to the recording chamber where it was continuously superfused with oxygenated ACSF at 33–35°C at the rate of 3–4 ml min^−1^.

### Electrophysiological recordings

Electrophysiological experiments were performed from CA3 principal cells or stratum radiatum interneurons in the CA3 area using the cell-attached and the whole-cell configuration of the patch-clamp technique in current or voltage-clamp mode. Neurons were visualized using an upright microscope (Olympus BX51WI) equipped with differential interference contrast (DIC) optics and infrared video camera.

Patched electrodes were pulled from borosilicate glass capillaries (Hingelberg, Malsfeld, Germany). They had a resistance of 4–6 MΩ when filled with an intracellular solution containing (in mM): KCl 140, MgCl_2_ 1, EGTA 0.5, HEPES 10, Mg ATP 4, (pH 7.3; the osmolarity was adjusted to 290 mOsmol). Glutamatergic postsynaptic currents were recorded using patch pipettes filled with a solution containing the following (in mM): 120 Cs-MeSO4, 20 KCl, 10 HEPES, 0.5 EGTA, 0.3 Na-GTP, and 4 Mg-ATP 4, pH 7.2 (osmolality, 275–280 mOsm).

Recordings were made with a patch clamp amplifier (Axopatch 200A; Molecular Device, Sunnyvale, CA, USA). The stability of the patch was checked by repetitively monitoring the input and series resistance during the experiment. Cells exhibiting >15 changes in series resistance were excluded from the analysis. The series resistance was <25 MΩ and was not compensated.

Spontaneous glutamatergic and GABAergic postsynaptic currents were routinely recorded from a holding potential of −70 mV in the presence of bicuculline (10 μM) and DNQX (20 μM), respectively. Miniature currents were recorded in the presence of TTX (1μM) to block sodium currents and propagated action potentials. Spontaneous firing was recorded in the cell attached mode from CA3 principal cells and stratum radiatum interneurons.

### Drugs

Drugs used were: tetrodotoxin (TTX, purchased from Latoxan, Valence, France), 6,7-dinitroquinoxaline-2,3-dione (DNQX), bicuculline methiodide, biocytin, citalopram hydrobromide, asenapine maleate, WIN55,212-2 mesylate all purchased from Tocris (Tocris Cookson Inc., UK); fluoxetine (gift of Prof. L. Maffei, Pisa).

All drugs were dissolved in ACSF except DNQX that was dissolved in DMSO. The final concentration of DMSO in the bathing solution was 0.1%. At this concentration, DMSO alone did not modify the shape or the kinetics of synaptic currents. Reagents were prepared as stock solutions and stored before use as aliquots in tightly sealed vials at the recommended manufacturers' temperature. Drugs were applied in the bath via a three-way tap system, by changing the superfusion solution to one differing only in its drug(s) content). The ratio of flow rate to bath volume ensured complete exchange within 2 min.

### Data acquisition and analysis

Data were acquired and digitized with an A/D converter (Digidata, 1200, Molecular Device, Sunnyvale, CA, USA) and stored on a computer hard disk. Acquisition and analysis were performed with Clampfit 9 (Molecular Device, Sunnyvale, CA, USA). Data were sampled at 20 kHz and filtered with a cut off frequency of 2 kHz. The resting membrane potential (RMP) was measured immediately after break-in and establishing whole-cell recording. The membrane input resistance (R_in_) was calculated by measuring the amplitude of voltage responses to steady hyperpolarizing current steps of increasing intensity, using the Clampfit 10.0 program (Molecular Device, Sunnyvale, CA, USA).

Spontaneous action potential-dependent and independent (miniature) AMPA and GABA_A_-mediated postsynaptic currents were analyzed using Clampfit 10.0 (Axon Instruments). This program uses a detection algorithm based on a sliding template. The template did not induce any bias in the sampling of events because it was moved along the data trace by one point at a time and was optimally scaled to fit the data at each position. The detection criterion was calculated from the template-scaling factor and from how closely the scaled template fitted the data.

Unless otherwise stated, data are presented as mean ± SEM. Significance of differences was assessed by Student's *t*-test, or Wilcoxon signed rank test, as appropriate. A *p* value < 0.05 was considered as significant.

## Results

### Fluoxetine reduces the frequency of GDPs

We first tested the effects of different concentrations of fluoxetine on spontaneous network-driven events which represent a hallmark of developmental circuits (Ben-Ari et al., 2012). We focused on the CA3 region of the hippocampus, because this region is centrally involved in GDPs generation (Menendez de la Prida et al., 1998; Bonifazi et al., 2009; Picardo et al., 2011; Allene et al., 2012). Here, GABAergic interneurons with large axonal arborizations operate as functional hubs able to synchronize large ensembles of cells (Bonifazi et al., 2009).

**Figure 1:**
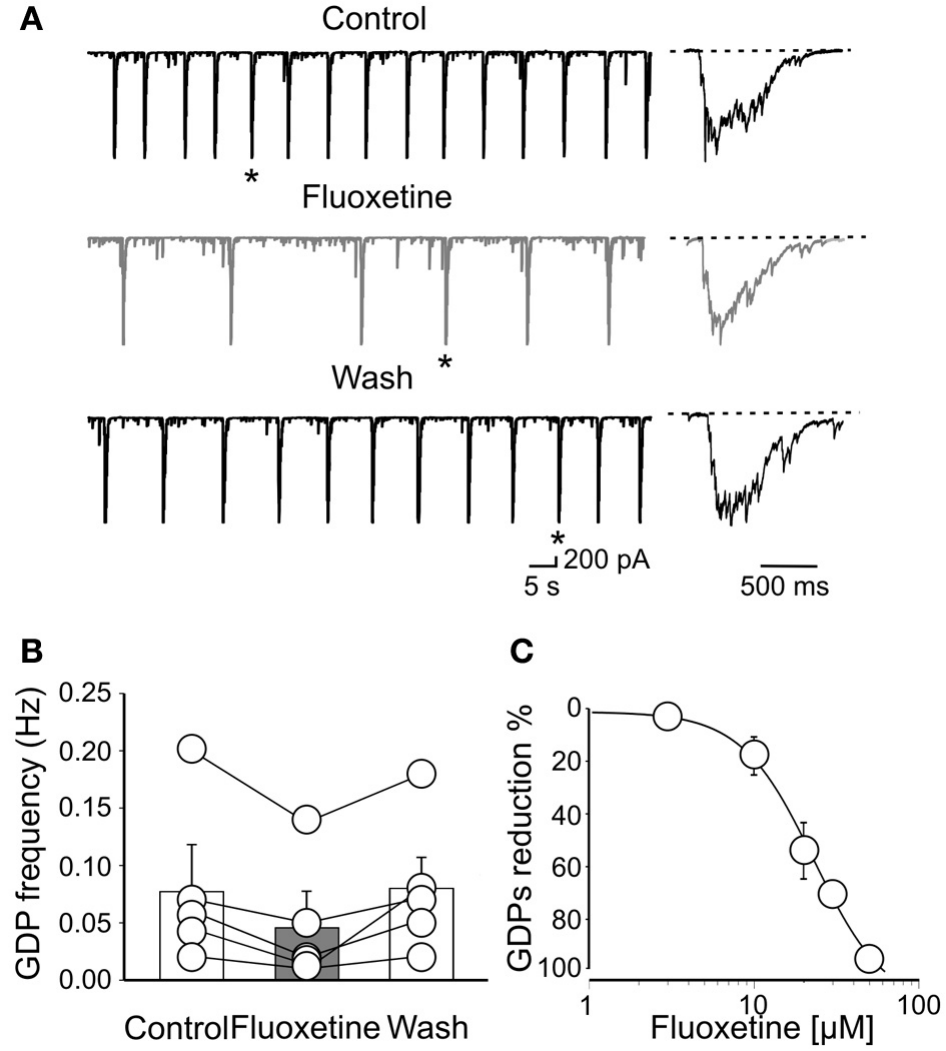
**Fluoxetine reduces the frequency of Giant Depolarizing potentials. (A)** GDPs recorded before, during and after (Wash) application of fluoxetine (20 μM). GDPs marked with an asterisk are shown on the right on an expanded time scale. **(B)** Population graph of GDPs frequency before, during and after application of fluoxetine. Columns represent the mean ± SEM. **(C)** Fluoxetine depressed GDPs in a concentration-dependent way. Each point represents the mean value of five individual experiments. Bars are the SEM (they are often within the symbols). Data points were fitted with the Hill equation (the IC_50_ value was 22 μ M).

GDPs occurred at the frequency of 0.08 ± 0.04 Hz (*n* = 5). As illustrated in Figures 1A,B, bath application of fluoxetine (20 μM), significantly reduced GDPs frequency (from 0.08 ± 0.04 Hz to 0.045 ± 0.031 Hz; *n* = 5; *p* = 0.021; Figure 1B) without altering their shape (Figure 1A). Fluoxetine did not affect the RMP (49 ± 3 mV and 49 ± 2 mV; *n* = 6; *p* = 0.2, before and during the administration of the drug), or the input resistance (475 ± 38 MΩ and 490 ± 41 MΩ, *n* = 6; *p* = 0.87), before and during the administration of the drug) of the recorded neurons. An almost complete recovery was obtained 11 ± 4 min after fluoxetine was washed out. The effect of fluoxetine on GDPs was concentration dependent. Exposure of individual slices to increasing concentrations of fluoxetine (from 3 to 50 μM) progressively reduced the frequency of GDPs. While at the concentration of 1 μM, known to efficiently block monoamine reuptake (Kobayashi et al., 2008), fluoxetine failed to affect correlated activity, at the concentration of 50 μM it completely abolished GDPs. As shown in cumulative dose-response curve of Figure 1C, fitting the experimental points with the Hill equation gave an IC_50_ value of 22 μM. This value is close to that obtained after chronic administration of the antidepressant which accumulates in the brain (Henry et al., 2005). Therefore in the following experiments we used a concentration of fluoxetine of 20 μM, close to the IC_50_ value.

**Figure 2:**
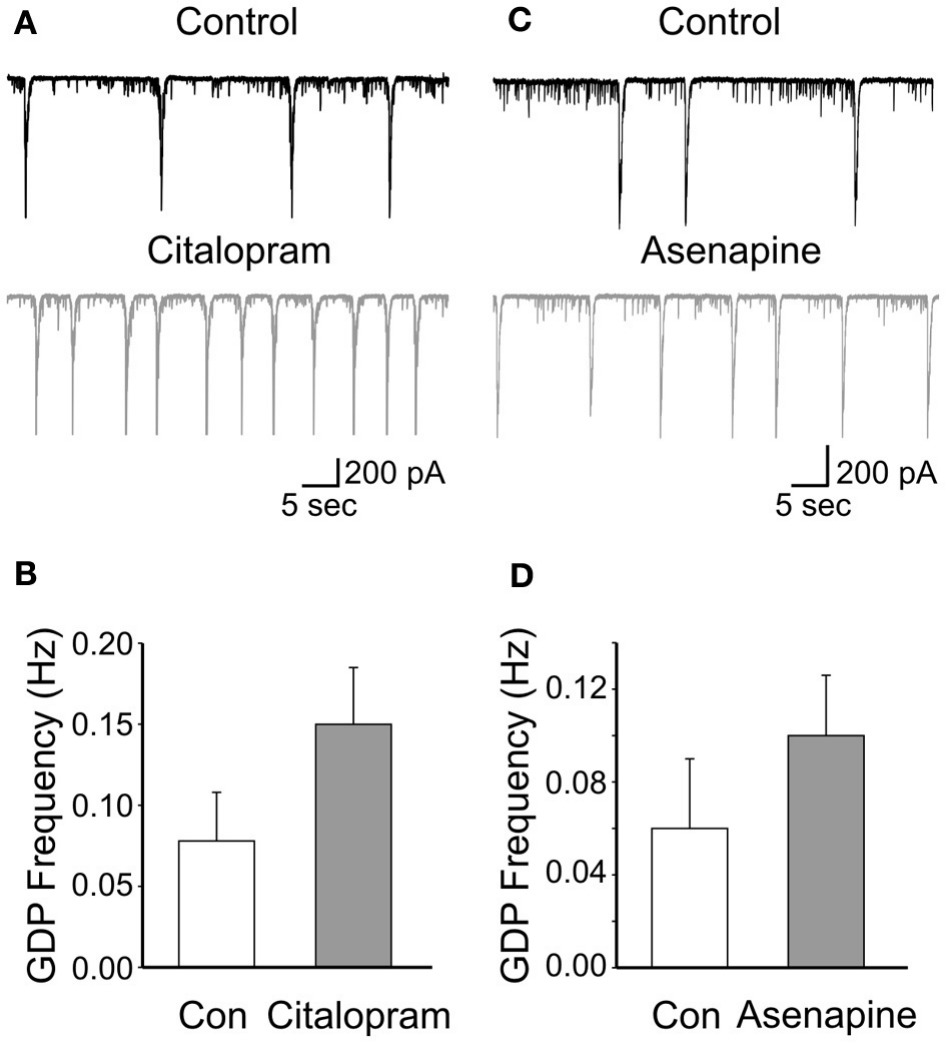
**Low concentrations of citalopram and asenapine enhance GDPs frequency. (A)** GDPs recorded before and during application of citalopram (1 μM). **(B)** Summary data for the results shown in A (*n* = 5; *p* < 0.001). Each column represents the mean ± SEM. **(C,D)** as for **(A,B)** but for asenapine (0.2 μM; *n* = 5; *p* = 0.02).

To further elucidate whether the effects of fluoxetine on GDPs were mediated by the indirect action on serotonin, we occluded serotonin uptake with citalopram (1 μM; Inoue et al., 1996). This drug *per se* caused an increase of GDPs frequency (from 0.078 ± 0.03 Hz to 0.15 ± 0.035 Hz; *n* = 5; *p* < 0.001; Figures 2A,B), suggesting the existence of a tonic serotonin component.

**Figure 3:**
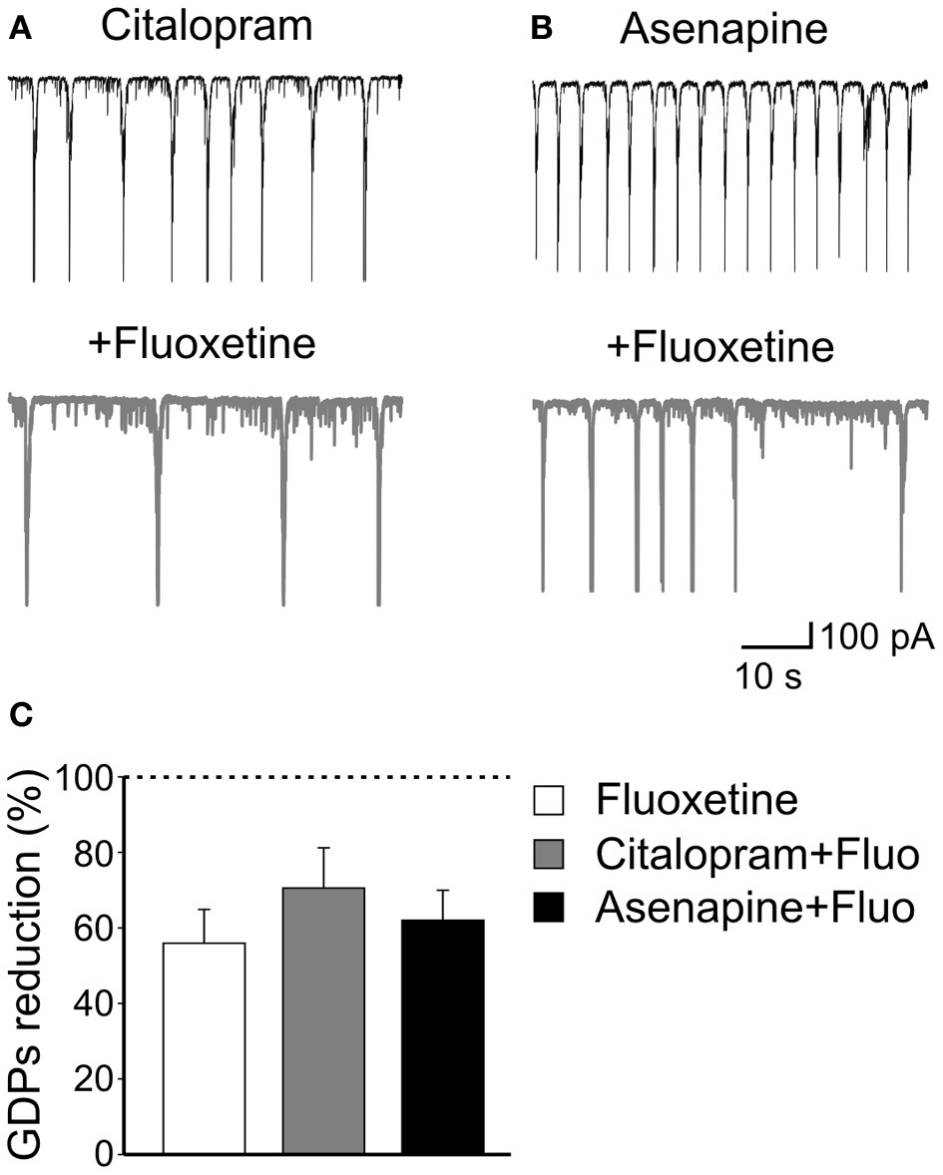
**The effects of fluoxetine on GDPs are independent of serotonin uptake or monoamine action. (A)** Citalopram, a selective serotonin reuptake inhibitor, at the concentration of 1 μM enhanced the frequency of GDPs. Addition of fluoxetine (20 μM) significantly reduced the frequency of GDPs. **(B)** Asenapine, a monoamine receptors blocker, also increased GDPs frequency that was reduced by subsequent addition of fluoxetine. **(C)** Summary data for the results shown in **(A)** and **(B)**. Fluoxetine-induced reduction of GDPs frequency was similar when the antidepressant was administered alone or together with citalopram or asenapine (*p* = 0.17).

However, after incubating the slices for 30 min with citalopram, addition of fluoxetine significantly reduced the frequency of GDPs of 70.8 ± 10.6%; *n* = 5; *p* = 0.02; (Figures 3A,C). This value was not significantly different from that obtained with fluoxetine alone (*p* = 0.3). Similarly, application of fluoxetine in the presence of asenapine (0.2 μM), a drug used for the acute treatment of schizophrenia as well as bipolar disorders which affects monoamine, histamine and muscarinic receptors (McIntyre, 2010), significantly reduced GDPs frequency in a way that was comparable to that found in the absence of the drug (reduction of 62 ± 8%; *n* = 5; *p* = 0.03; Figures 3B,C). Like citalopram, asenapine *per se* caused an enhancement of GDPs frequency (from 0.06 ± 0.03 Hz to 0.1 ± 0.026 Hz; *n* = 5; *p* = 0.02; Figures 2C,D).

These data clearly indicate that fluoxetine acts on GDPs independently of serotonin action since its effects persisted in the presence of citalopram, a selective serotonin uptake inhibitor and after pharmacological occlusion of monoamine receptors with asenapine.

### Fluoxetine enhances the frequency of miniature gabaergic but not glutamatergic currents

**Figure 4:**
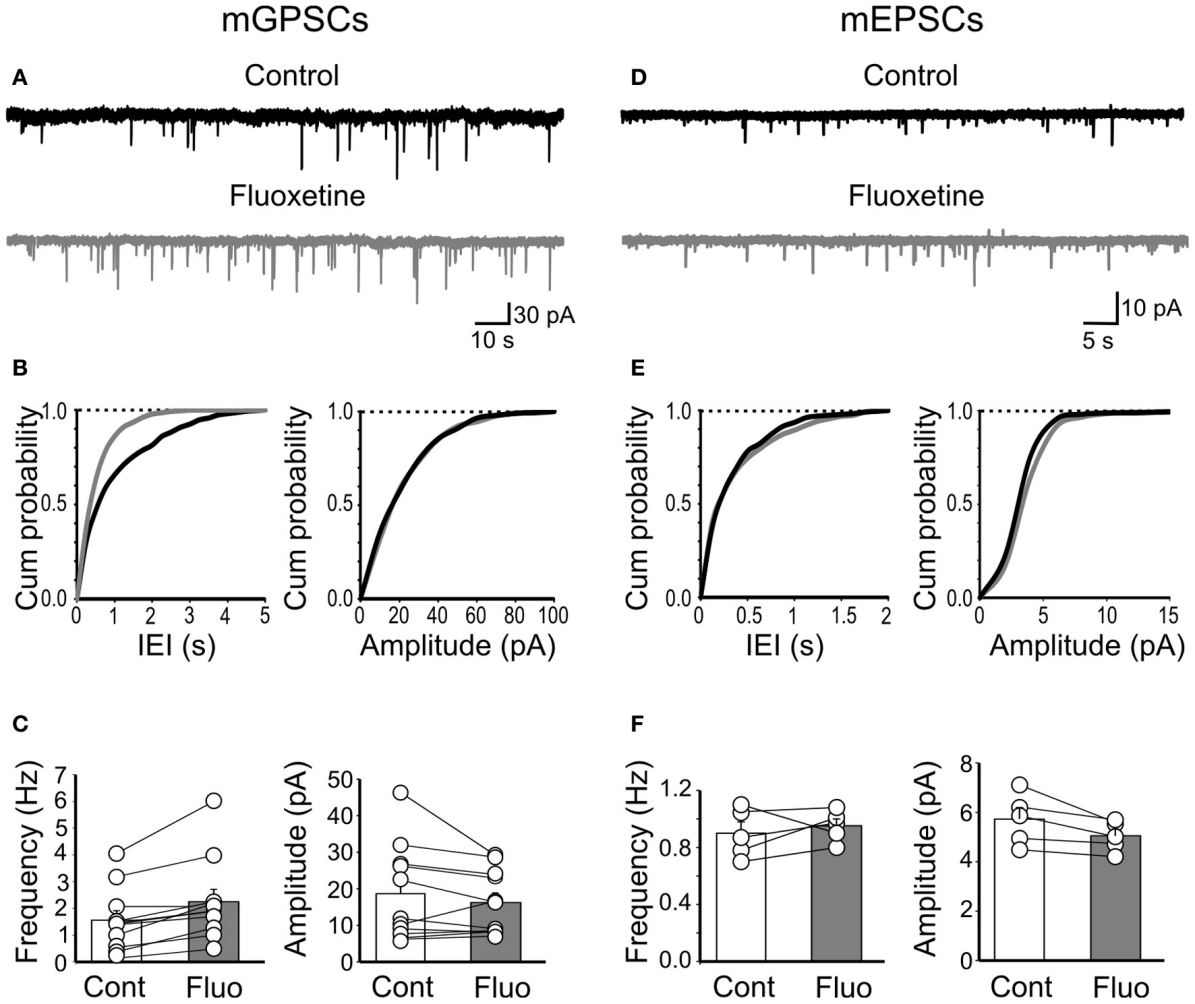
**Fluoxetine increased the frequency of miniature GPSCs but did not affect miniature EPSCs. (A)** Continuous voltage clamp recordings of mGPSCs from a holding potential of −60 mV in the presence of DNQX (20 μM), D-APV (10 μM), and TTX (1 μM) before and during application of fluoxetine (20 μM). **(B)** Cumulative distribution plots of inter event interval (IEI) and amplitude for the cell shown in A before (black) and during (grey) application of fluoxetine. **(C)** Summary plots of the mean frequency (*n* = 11; *p* = 0.002) and amplitude (*n* = 11; *p* = 0.17) of all cells tested (±SEM). **(D–F)** as in **(A–C)** but for mEPSCs (recorded in the presence of bicuculline, 10 μM, TTX, 1 μM). No significant differences in frequency (*n* = 5; *p* = 0.5) or amplitude (*n* = 5; *p* > 0.05) of mEPSCs were detected.

Since GDPs are generated by the synergistic action of GABA and glutamate, both of which depolarizing and excitatory (Ben-Ari et al., 1989; Cherubini et al., 1991), we next examined whether changes in spontaneous miniature GABA_A_- and AMPA-mediated postsynaptic currents (mGPSCs and mEPSCs, respectively) could account for the effects of fluoxetine on GDPs. Miniature GABAergic or glutamatergic currents were recorded in the presence of TTX (1 μM) and bicuculline or DNQX, respectively. Miniature GPSCs occurred at the frequency of 1.55 ± 0.35 Hz (*n* = 11). As shown in Figures 4A–C, application of fluoxetine (20 μM), significantly enhanced mGPSCs frequency (from 1.55 ± 0.35 Hz to 2.25 ± 0.46 Hz; *p* = 0.002) without affecting their amplitude (on average the mGPSCs amplitude was 18.6 ± 3.9 pA and 16.2 ± 2.6 pA, before and after fluoxetine, respectively; *p* = 0.17). This is clearly illustrated in the example of Figure 4A and in the cumulative inter event interval (IEI) plot of Figure 4B, where the distribution of mGPSCs obtained in the presence of fluoxetine is shifted to the left respect to those obtained in the absence of the drug (*p* < 0.05; K–S test) and in the summary graph of Figure 4C.

Fluoxetine did not alter the frequency or amplitude of mEPSCs. The frequency of mEPSCs was 0.9 ± 0.07 Hz and 0.9 ± 0.04 Hz, before and during fluoxetine, respectively (*n* = 5; *p* = 0.5; Figures 4D–F) and their amplitude (5.7 ± 0.46 pA and 5.04 ± 0.27 pA, before and during fluoxetine, respectively; *n* = 5; *p* > 0.05; Figures 4D–F). These results indicate that fluoxetine-induced changes in GDPs frequency probably involve GABAergic but not glutamatergic signaling.

**Figure 5:**
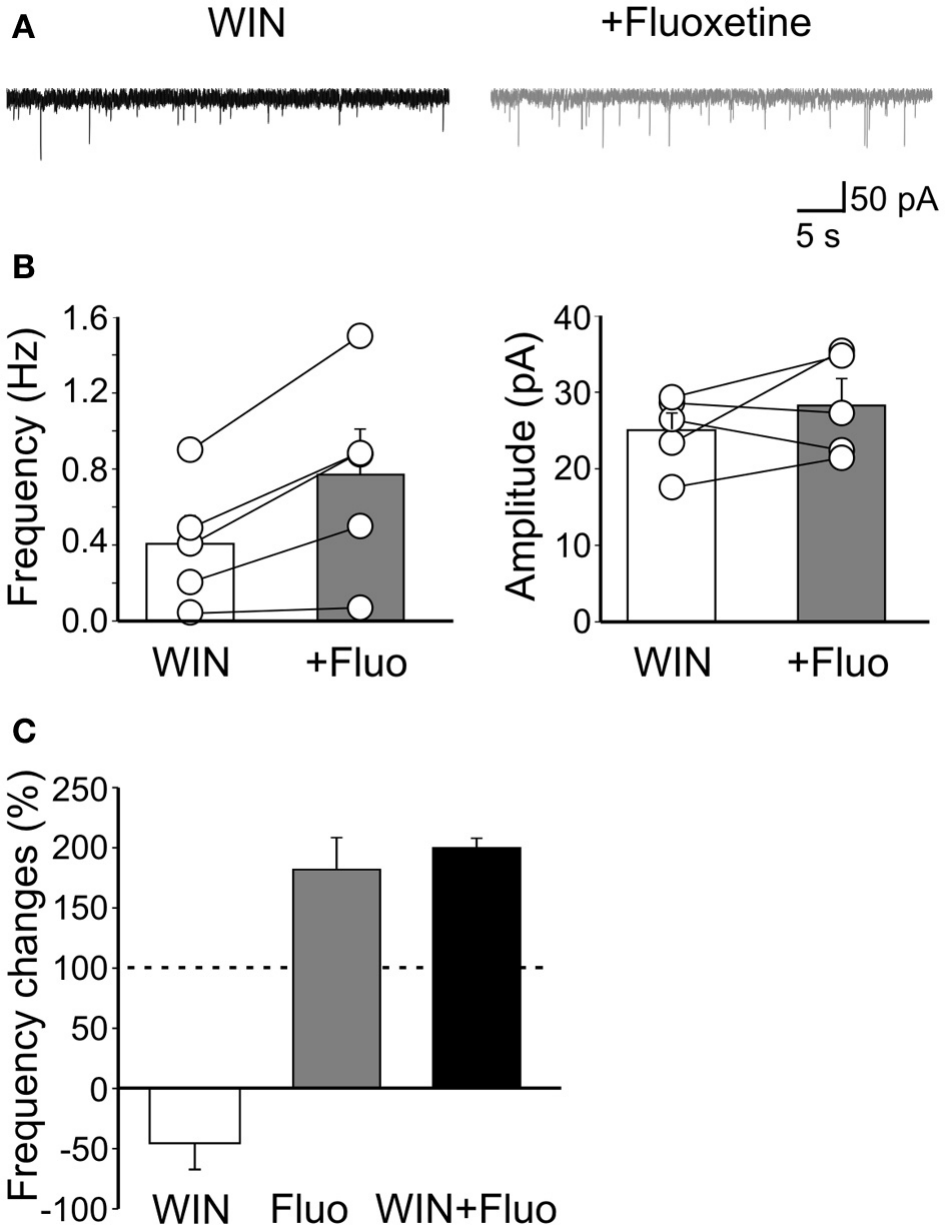
**Fluoxetine-induced increase of mGPSCs frequency does not involve CB1 receptor-positive GABAergic interneurons. (A)** Sample traces of mGPSCs recorded in the presence of WIN55,212-2 (1 μM) and WIN55,212-2 plus fluoxetine (20 μM). **(B)** Summary plots of the mean frequency (*n* = 5; *p* = 0.02) and amplitude (*n* = 5; *p* = 0.3) of mGPSCs recorded in the presence of WIN55,212-2 (white columns) and WIN55,212-2 plus fluoxetine (gray columns) for all cells tested (±SEM). **(C)** Frequency changes (as percentage of controls) induced by bath application of WIN55,212-2 (white), fluoxetine (grey) and fluoxetine plus WIN55,212-2 (black). Note that while WIN55,212-2 depressed the frequency of mGPSCs fluoxetine increased it. In addition, a similar percentage increase of mGPSCs frequency occurred when fluoxetine was applied alone or in the presence of WIN55,212-2.

Miniature events are generated mainly by spontaneous, action potential independent release of GABA from fast spiking, cannabinoid (CB) receptor-negative and regular spiking CB1 receptor-positive GABAergic interneurons. To evaluate whether fluoxetine selectively affects a particular interneuron population we used WIN55,212-2, a CB1/CB2 receptor agonist, to occlude cannabinoid receptors present on axon terminals of cholecystokinin-positive regular spiking cells. In our hands, WIN55,212-2 was acting on CB1 receptors as demonstrated by a previous study from the CA3 hippocampal region of newborn rats and mice (Caiati et al., 2012). WIN55,212-2 (1 μM) *per se*, caused a reduction of mGPSCs frequency of 46.2 ± 21.8% (*n* = 5; *p* < 0.05) in the absence of any change in amplitude. Bath application of AM 251 (5 μM), a selective CB1 receptor antagonist, did not produce any effect on mGPSCs amplitude (101 ± 3.2% of controls; *n* = 5; *p* > 0.05) and frequency (104 ± 8.5% of controls; *n* = 5; *p* > 0.05), indicating that CB1 receptors were not tonically active. Addition of fluoxetine to slices incubated for 20–30 min in the presence of WIN55,212-2, enhanced the frequency of GPSCs (from 0.40 ± 0.14 Hz to 0.77 ± 0.23 Hz; *n* = 5; *p* = 0.02) without significantly modifying their amplitude (25.03 ± 2.1 pA and 28.2 ± 3.5 pA, *n* = 5; before and during WIN55,212-2, respectively; *p* = 0.3; Figure 5).

Therefore, while WIN55,212-2 reduced mGPSCs frequency, fluoxetine enhanced it (Figure 5C). It should be stressed that the percentage increase of GPSCs frequency detected when fluoxetine was applied after WIN55,212-2 was similar to that obtained when the drug was applied in the absence of WIN55,212-2, indicating that fluoxetine acts mainly on CB1 receptor-negative GABAergic interneurons (*p* = 0.2; Figure 5C). These results further stress previous data obtained from the CA1 hippocampal region of juvenile animals, showing a selective effect of fluoexitine on GABAergic neurotransmission from fast spiking CB1 receptor-negative interneurons (Méndez et al., 2012).

It is worth mentioning that the effects of fluoxetine on mGPSCs were independent of serotonin action since, in the presence of citalopram, fluoxetine still enhanced mGPSCs frequency of 186 ± 29% (*n* = 5; *p* = 0.01; data not shown).

### Fluoxetine reduces the frequency and amplitude of spontaneously occurring GPSCs

**Figure 6:**
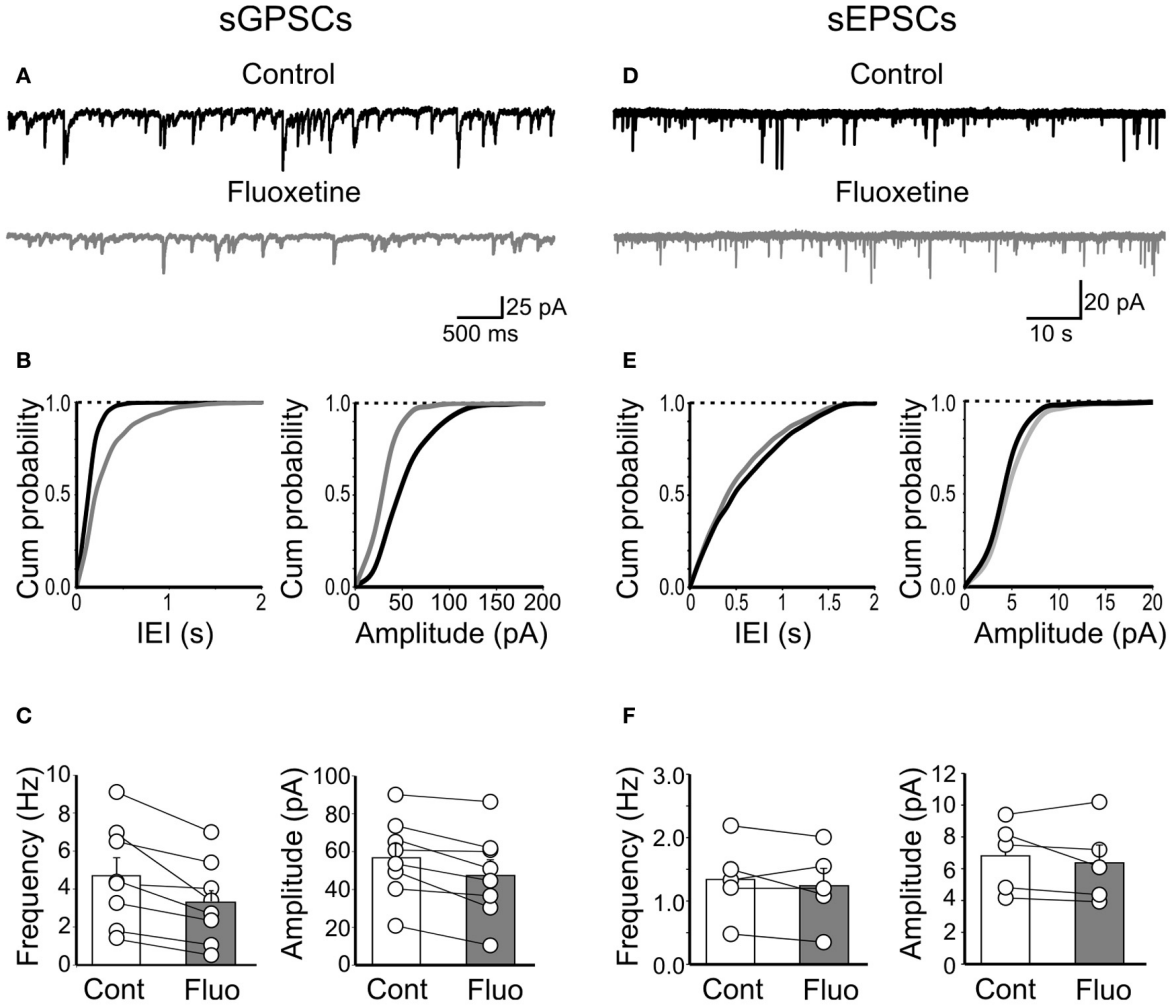
**Fluoxetine reduced the frequency and amplitude of spontaneous action potential-dependent GPSCs but did not affect AMPA-mediated EPSCs. (A)** Continuous voltage clamp recordings of sGPSCs detected at −60 mV in the presence of DNQX (20 μM) and D-APV (10 μM), before and during application of fluoxetine (20 μM). **(B)** Cumulative distribution plots of inter event interval (IEI) and amplitude for the cell shown in **(A)** before (black) and during (grey) application of fluoxetine. **(C)** Summary plots of the mean frequency (*n* = 8; *p* = 0.007) and amplitude (*n* = 8; *p* = 0.004) of all cells tested (±SEM). **(D–F)** As in **(A–C)** but for sEPSCs. No significant differences in frequency (*n* = 5; *p* = 0.4) or amplitude (*n* = 5; *p* = 0.38) were detected.

Next, we tested the effects of fluoxetine on the frequency and amplitude of spontaneous action potential-dependent GABAergic (sGPSCs) and glutamatergic (sEPSCs) currents. Unlike mGPSCs, fluoxetine (20 μM) significantly reduced the frequency (from 4.7 ± 0.9 Hz to 3.3 ± 0.76 Hz; *n* = 8; *p* = 0.007) and the amplitude (57 ± 7 pA to 47 ± 8 pA; *n* = 8; *p* = 0.004; Figures 6A–C) of sGPSCs, with a relative loss of larger amplitude events (>60 pA). The reduction in amplitude of spontaneous events was not associated with changes in deactivation kinetics, since similar decay time values were obtained for sGPSCs recorded in the absence or in the presence of the antidepressant (15.3 ± 0.9 ms and 16.7 ± 0.7, in the absence and in the presence of fluoxetine, respectively; *n* = 9; *p* = 0.2).

In contrast, fluoxetine failed to affect sEPSCs. The frequency of sEPSCs was 1.34 ± 0.27 Hz and 1.24 ± 0.27 Hz, before and during fluoxetine application (*n* = 5; *p* = 0.4; Figures 6D–F) and the amplitude was 7 ± 1 pA and 6 ± 1 pA before and during fluoxetine application (*p* = 0.38; Figures 6D–F).

These data further confirm a selective action of fluoxetine on GABAergic interneurons.

**Figure 7:**
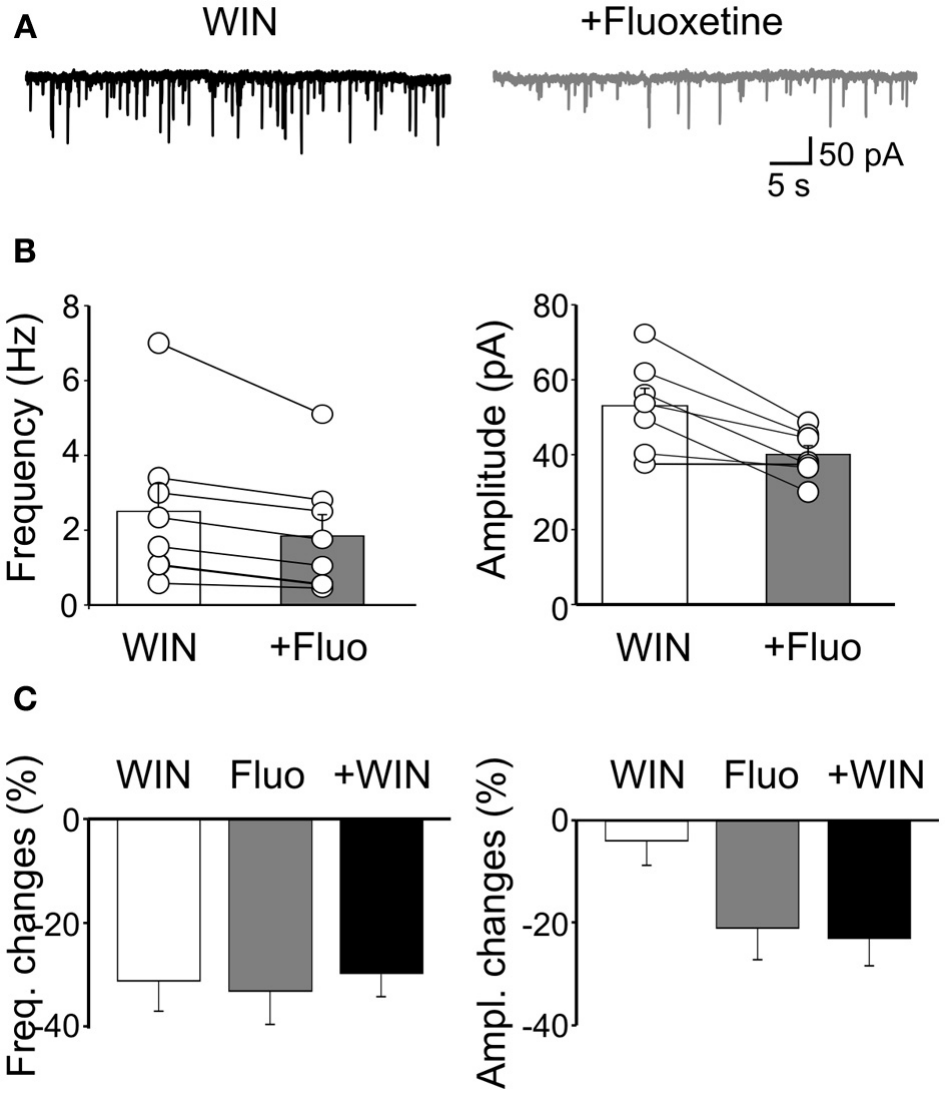
**Fluoxetine-induced reduction of sGPSCs frequency and amplitude does not involve CB1 receptor-positive GABAergic interneurons. (A)** Sample traces of sGPSCs recorded in the presence of WIN55,212-2 (1 μM) and WIN55,212-2 plus fluoxetine (20 μM). **(B)** Summary plots of the mean frequency (*n* = 8; *p* = 0.008) and amplitude (*n* = 7; *p* = 0.008) of sGPSCs recorded in the presence of WIN55,212-2 (white columns) and WIN55,212-2 plus fluoxetine (gray columns) for all cells tested (±SEM). **(C)** Frequency and amplitude changes (as percentage of controls) induced by bath application of WIN55,212-2 (white), by fluoxetine (grey) and fluoxetine plus WIN55,212-2 (black). Note that while WIN55,212-2 depressed only the frequency of sGPSCs, fluoxetine depressed both the frequency and the amplitude. In addition, fluoxetine depressed to a similar extent the amplitude and the frequency of sGPSCs when applied alone or in the presence of WIN55,212-2.

To evaluate whether the effects of fluoxetine on sGPSCs did not involve CB receptor-positive GABAergic interneurons, we applied WIN55,212-2 (1 μM). This drug significantly reduced the frequency of sGPSCs of 31.6 ± 6% (*n* = 7; *p* < 0.001) but not their amplitude (4 ± 4.8%; *p* = 0.4; Figure 7C). Then, in another set of experiments, we incubated the slices for 20–30 min in the presence of WIN55,212-2 (1 μM). Application of fluoxetine in the presence of WIN55,212-2 further decreased sGPSCs frequency (from 2.5 ± 0.7 Hz to 1.8 ± 0.56 Hz; *n* = 8; *p* = 0.008) and reduced their amplitude (from 53 ± 4.5 pA to 39.9 ± 2.4 pA; *p* = 0.008; Figure 7) with a selective loss of higher amplitude events.

The similar reduction of sGPSCs frequency observed when fluoxetine was applied alone or in the presence of WIN55,212-2 and the reduction of amplitude detected only when fluoxetine was added to WIN55,212-2, clearly indicate that fluoxetine and WIN55,212-2 act on two different neuronal GABAergic populations.

Fluoxetine effects on sGPSCs did not involve serotonin, since in the presence of citalopram (1 μM) the drug still caused a significant reduction of both sGPSCs frequency (55.5 ± 12%; *n* = 5; *p* < 0.05) and amplitude (36 ± 4%; *n* = 5; *p* < 0.05; data not shown).

### Fluoxetine reduces the firing rate of gabaergic interneurons and principal cells

**Figure 8:**
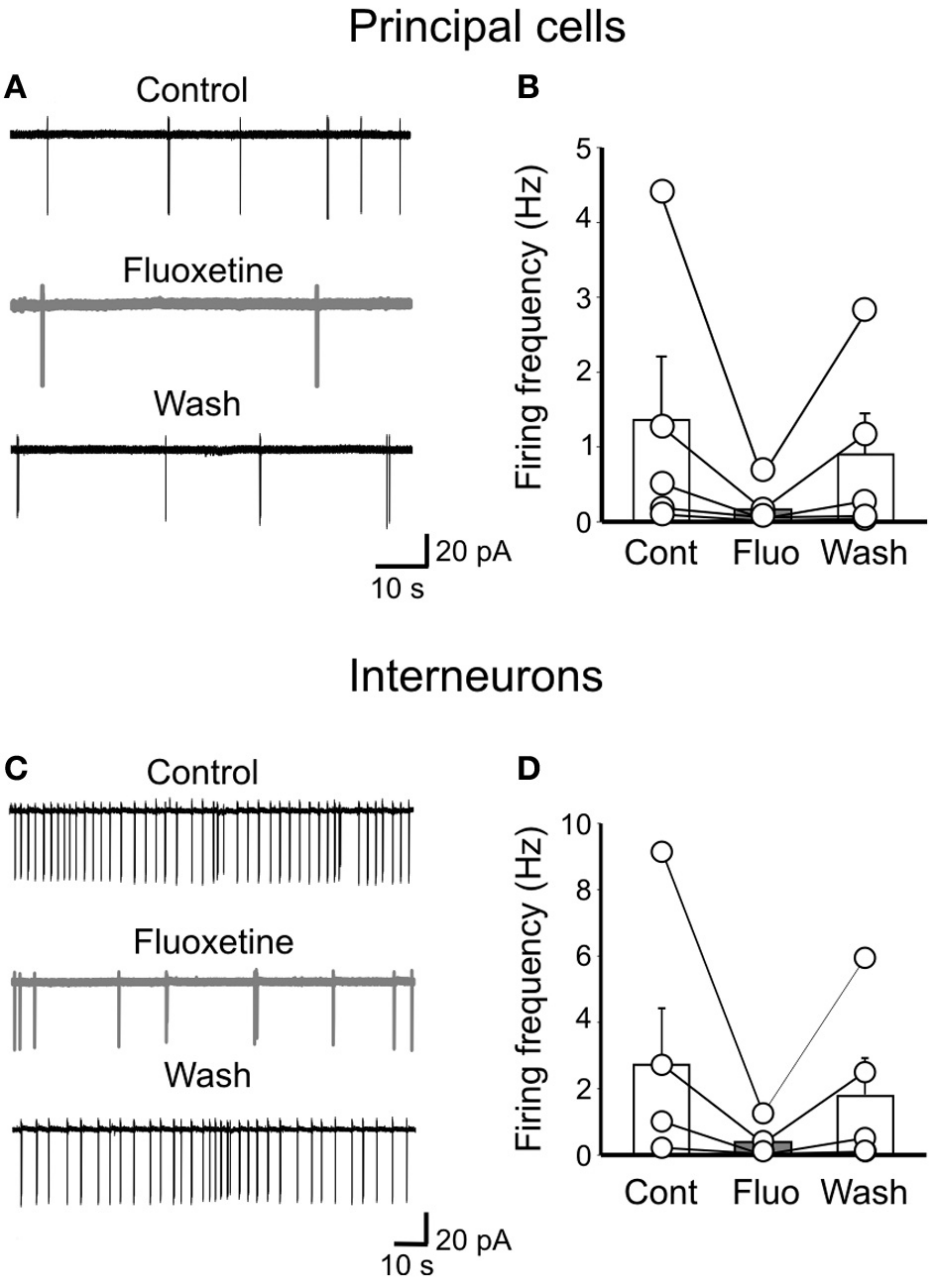
**Fluoxetine reduces the firing rate of principal cells and interneurons. (A)** cell attach recordings of a pyramidal cell before, during and after (Wash) application of fluoxetine (20 μM). **(B)** Population graph of the firing frequency of pyramidal cells (*n* = 5) before (Cont), during (Fluo) and after application of fluoxetine (Wash). Columns represent the mean, bars the SEM. The effects of fluoxetine were significantly different from controls (*p* < 0.05). **(C,D)** as in **(A,B)** but for GABAergic interneurons in stratum radiatum (*n* = 5; *p* < 0.05).

In analogy with the synchronized activity generated in the disinhibited hippocampus (De la Prida et al., 2006), GDPs emerge when a sufficient number of cells fire and the excitability of the network attains a certain threshold within a restricted time window (Ben-Ari et al., 2012). Although the entire hippocampal network possesses the capacity to generate GDPs, the CA3 area is particularly well equipped because of its extensive glutamatergic recurrent collaterals and spontaneous intrinsic bursts that can drive other neurons to fire (Sipilä et al., 2005; Safiulina et al., 2008). Therefore, in cell attach experiments (to preserve the intracellular milieu of the recorded neurons), we measured the spontaneous firing rate of stratum radiatum GABAergic interneurons and pyramidal cells. Bath application of fluoxetine reversibly slowed down the firing rate of both principal cells (from 1.57 ± 0.74 Hz to 0.56 ± 0.37 Hz; *n* = 5; *p* < 0.05; Figures 8A,B) and interneurons (from 2.7 ± 1.7 Hz to 0.33 ± 0.22 Hz; *n* = 5; *p* < 0.05; Figures 8C,D).

The fluoxetine-induced reduction of spontaneous firing may severely alter network excitability and GDPs generation.

## Discussion

The present results clearly indicate that the acute exposure of immature hippocampal slices to fluoxetine, a major antidepressant widely used for the treatment of mood disorders, severely alters GABA_A_-mediated network activity under the form of GDPs which, as already mentioned, represent a hallmark of developmental circuits. This effect appears to be dependent on the reduced release of GABA from subpopulations of GABAergic interneurons not expressing CB1 receptors.

The effects of fluoxetine on GDPs were not indirectly mediated *via* serotonin or other amines known to be the targets of the antidepressant, since they persisted following occlusion of serotonin uptake with citalopram or blockade of monoamine, histamine and muscarinic receptors with asenapine. Unlike fluoxetine, both citalopram and asenapine enhanced GDPs frequency probably by directly interfering with “ambient” serotonin present at low levels in the extracellular space. Interestingly, the concentration of fluoxetine needed to affect GABA release was higher than that necessary to impair serotonin transporters (Kobayashi et al., 2008), suggesting a concentration-dependent effect on different targets. The concentration we used (close to the IC_50_ value), could be clinically relevant since, due to a very slow elimination (Henry et al., 2005), during chronic treatment the drug accumulates in the brain reaching a concentration 20 times higher than that present in the plasma (Karson et al., 1993).

GDPs are generated by the synergistic action of GABA and glutamate, both of them depolarizing and excitatory (Bolea et al., 1999; Ben-Ari et al., 2012) and therefore, their depression by fluoxetine may involve changes of glutamatergic, GABAergic synaptic transmission or both. We can exclude the involvement of glutamate, since spontaneous AMPA-mediated EPSCs were not affected by the drug. It is therefore likely that major targets of the antidepressant are GABAergic interneurons. Fluoxetine was recently shown to reduce GABA release from PV-positive fast spiking GABAergic interneurons in the hippocampus of juvenile animals (Méndez et al., 2012). Although PV-positive basket cells certainly contribute to the spontaneous action potential-dependent release of GABA (Freund and Katona, 2007) we cannot exclude the participation of other interneuron subtypes. However, the similar degree of depression in GPSCs amplitude and frequency observed when slices were exposed to fluoxetine or fluoxetine plus WIN55,212-2, indicates, in line with the results of Méndez et al. (2012), that CB1-positive interneurons were not affected.

Fluoxetine has been reported to positively modulate *via* a novel allosteric site GABA_A_ receptors (Robinson et al., 2003). However, in the present case this is unlikely since the drug exerted a depressant and not a potentiating action on the frequency and amplitude of spontaneous GPSCs. In addition, the lack of fluoxetine-induced changes in the deactivation kinetics of spontaneous events allow excluding a direct effect of the drug on the gating properties of GABA_A_ receptors. The depressant effect of fluoxetine is probably presynaptic and may reflect the preferential loss of higher amplitude events following the impairment of synchronous release of GABA from distinct release sites. At the concentration used, the drug may inhibit vesicle exocytosis (Henkel et al., 2010) by interfering with calcium entry through P/Q (Pfenninger et al., 2003) or other types of calcium channels (Deák et al., 2000; Traboulsie et al., 2006). This may lead to a reduced cell excitability. The reduction of spontaneous firing rate of principal cells and interneurons in cell attach experiments strengthens this hypothesis. Fluoxetine may affect cell excitability also by inhibiting voltage-gated sodium (Lenkey et al., 2006; Igelström and Heyward, 2012) and potassium channels (Yeung et al., 1999). Although inhibition of potassium currents should enhance cell excitability, in some cases (i.e., impairment of the delayed rectifier) it may prevent membrane potential from returning to a level where sodium channels can de-inactivate, thus reducing the availability of sodium channels and repetitive firing. This mechanism has been suggested to underline the action of the antiepileptic drug levetiracetam (Madeja et al., 2003).

As already mentioned, GDPs emerge when a sufficient number of cells fire and the excitability of the network attains a certain threshold within a restricted time window. Therefore, it is reasonable to believe that a reduced neuronal excitability may lead to a reduction of cell firing, network desynchronization and GDPs depression.

It is worth mentioning that, in the present experiments, fluoxetine exerted opposite effects on spontaneous calcium-independent (miniature) and calcium-dependent release of GABA. While we cannot exclude the possibility that the antidepressant controls mini and spontaneous action potential-dependent events through different mechanisms, as a unifying view we can hypothesize that the elevated extracellular levels of GABA, following the asynchronous release may reduce cell excitability and synchronicity *via* shunting inhibition. A similar perspective has been put forward by Méndez et al. (2012) to explain the fluoxetine-induced increase of miniature GABAergic currents associated with a reduction of GABA release from fast spiking basket cells. According to these authors, the synergistic reduction of synchronous release from fast spiking cells and the enhanced spontaneous asynchronous release of GABA can deteriorate spike-timing precision and γ oscillations. However, in our case, this is unlikely since, early in postnatal life, ambient GABA exerts a depolarizing and excitatory action on target cells (Marchionni et al., 2007). Therefore, fluoxetine-induced elevation of the extracellular GABA levels should be associated with an increase and not a decrease in cell excitability.

Whatever is the mechanism, it is clear that fluoxetine severely affects correlated network activity in the developing hippocampus. Since calcium transients associated with GDPs act as coincident detector signals for enhancing synaptic efficacy at emerging glutamatergic (Mohajerani et al., 2007) and GABAergic synapses (Kasyanov et al., 2004), a disruption of GDPs may have serious consequences on the construction and functional organization of the hippocampal networks.

Although the present experiments do not directly demonstrate whether the chronic maternal treatment with fluoxetine alters neuronal circuits and brain development in pups, nonetheless, they highlight the crucial role of the antidepressant on GABAergic signaling in the immature hippocampus. While further experiments in pups from chronically treated mothers would help clarifying this point, considering the crucial role of GABA in the developing networks, caution should be taken in treating pregnant woman affected by mood disorders with fluoxetine.

## Author contributions

Enrico Cherubini and Maddalena D.Caiati: conceived and designed the experiments. Maddalena D.Caiati: performed the experiments, analyzed data. Enrico Cherubini: wrote the paper. Both authors approved the final version of the manuscript.

### Conflict of interest statement

The authors declare that the research was conducted in the absence of any commercial or financial relationships that could be construed as a potential conflict of interest.
